# Neuropsychological rehabilitation interventions for people with an acquired brain injury. A protocol for a systematic review of economic evaluation

**DOI:** 10.12688/hrbopenres.13144.2

**Published:** 2020-12-08

**Authors:** Eileen Mitchell, Elayne Ahern, Sanjib Saha, Dominic Trepel

**Affiliations:** 1 Global Brain Health Institute, Trinity College Dublin, Dublin, Ireland and University of California, San Francisco, USA; 2 School of Biological Sciences, IGFS, Gibson Institute, Queen's University Belfast, Belfast, Northern Ireland, UK; 3 Department of Psychology, University of Limerick, Limerick, Ireland; 4 Health Economics Unit Department of Clinical Science (Malmö), Lund University, Malmö, Sweden; 5 School of Medicine, Trinity College Dublin, University of Dublin, Dublin, Ireland

**Keywords:** Systematic review, Protocol, Acquired brain injury, Economic evaluation, cost-effectiveness

## Abstract

**Background:** New emerging evidence has demonstrated the need for effective interventions to help people living with an acquired brain injury (ABI). Evidence on cost-effectiveness, which can help inform use of limited resources, is scarce in this area and therefore the purpose of this systematic review is to critically appraise and consolidate the current evidence on economic evaluations of ABI rehabilitation interventions.

**Methods: **Systematic review methodology will be applied to identify, select and extract data from published economic evaluation studies (trial-based, non-trial based, simulation-based, decision model and trial-based model economic evaluations) of ABI treatment interventions in adults. A systematic literature search will be conducted on the following electronic databases: EMBASE, Econlit, CINAHL, Medline, the National Health Service Economic Evaluation Database and PsyclNFO. This review will only include cost-effectiveness analysis studies (e.g., cost per life year gained), cost-benefit and cost minimisation analyses in which the designs were randomised controlled trials (RCTs), non-RCT studies, cost-utility analyses (e.g., cost per quality-adjusted life year (QALY) gained or cost per disability-adjusted life year averted), cohort studies, and modelling studies. Only studies that were published in the english language, associated with adults who have an ABI will be included. There will be no restrictions on perspective, sample size, country, follow-up duration or setting. The search strategy terms will include the following: acquired brain injury, brain*; cost*; or cost–benefit analysis*. Following data extraction, a narrative summary and tables will be used to summarize the characteristics and results of included studies.

**Discussion:** The findings from this review will be beneficial to health policy decision makers when examining the evidence of economic evaluations in this field. In addition, it is anticipated that this review will identify gaps in the current economic literature to inform future-related research.

**Systematic review registration:** PROSPERO CRD42020187469 (25 ^th^ June 2020).

## Introduction

Acquired brain injury (ABI) is an injury to the brain that is not hereditary, degenerative, congenital, or brought about by birth trauma; it happens after birth ^1^. ABI includes both brain injuries with a non-traumatic cause (such as brain tumours and stroke) and with a traumatic cause (such as automobile crashes and falls) ^2^. In Ireland, there does not currently exist an adequate system for registering the prevalence and incidence and of ABI on an international and national basis, but informal estimates from the Irish National Audit of Stroke Care and data from the Hospital In-Patient Enquiry scheme indicates that each year in the country around 20,000 brain injuries occur ^3^. ABI is the third most common cause of disability and death internationally each year, and can result in long-lasting health, economic and social effects on an individual, their family and friends and health systems ^4, 5^.

The economic consequences of having an ABI for both individuals and society are substantial and often problematic to estimate precisely. In the USA, ABI has been estimated to cost around €76.5 billion per year ^6^. Whilst no official costs of ABI exists in Europe, the costs of traumatic brain injury (TBI) are projected to be €33 billion (around USD $45.4 billion) ^7^, additionally, it is estimated that stroke care treatment are around €27 billion, with around €18.5 billion contributing towards the direct medical costs and around €8.5 billion for indirect costs (e.g., absence from work due to illness, loss of productivity) ^8^. The evidence indicates that rehabilitation interventions after having an ABI improve health outcomes, increase quality of life and survival probabilities as well as help decrease costs by shortening hospital stays ^9^. There is currently no available cure for a person living with an ABI, only symptom relieving therapies, which include either pharmacological (use of drugs) ^10^ or non-pharmacological ^11^. The vast number of non-pharmacological interventions for patients with ABI disorders have risen in recent years ^12, 13^ and many have been shown to be effective in reducing behavioural and psychological symptoms associated with having an ABI. Research suggests that effective non-pharmacological rehabilitation interventions include cognitive simulation, physical exercise and behavioural and occupational therapy, targeting persons with ABI disorders separately and jointly ^14, 15^.

Whilst a wide range of available and generally effective non-pharmacological rehabilitation interventions are available, given that resources are limited and health and social care budgets are strained, it is imperative that every rehabilitation intervention can demonstrate evidence of effectiveness ^16^.

Moreover, the relative levels of benefit should be at a reasonable compared to the cost of the interventions, meaning, that rehabilitation interventions are cost-effective and that the health and social care services are getting the value for money from their healthcare budgets ^17^. For policy makers, having this information is vital when allocating scarce health care resources.

Economic evaluation (EE) is an analytical technique which measures, values, identifies and attempts to compare the cost and outcomes of two or more alternative programs or interventions. Economic evidence is gradually being recognized as an important decision making input tool for policy makers, ensuring that resources are used in a way that maximizes the benefits ^18, 19^. Unfortunately, there is a limited amount of consolidated economic evidence to guide the commissioning and improvement of services that support life after an ABI ^4, 20, 21^. Several systematic reviews which have specifically focused on the costs of stroke care have suggested the need for more economic evidence in the brain injury field ^22, 23^. A recent scoping review by Stolwyk *et al.* (2019) identified 30 studies of economic evaluations of neuropsychological rehabilitation for ABI recommended an urgent need to conduct more economic evaluations studies which examine rehabilitation following brain injury ^24^. Stolwyk and colleagues primarily aimed to describe the methodology used in economic evaluations of neuropsychological rehabilitation for ABI. Conclusions on the cost-effectiveness of neuropsychological rehabilitation was therefore limited from the scoping review of Stolwyk and colleagues as different standards of living and price levels between countries were not accounted for among the studies reviewed.

Therefore, the purpose of this systematic review is (i) critically to consolidate and literature on economic evaluations of neuropsychological rehabilitation for several types of ABIs including stroke, traumatic brain injury, or ABI from non-progressive aetiologies; formal comparisons will be made by adjusting costs to a reference year (ii) assess the quality of available evidence, and (iii) use the findings of this systematic review to identify additional areas needed for improving the conduct and reporting of economic evaluations in ABI ^25, 26^. Therefore, a systematic literature review of non-pharmacological rehabilitation economic evaluations in ABIs is a way to summarize available knowledge in the field, to identify common characteristics of the existing studies, assess current reporting standards in adherence to best practice guidelines and highlight areas where more research is required.

## Methods

This ABI systematic review protocol has been developed based on “The Preferred Reporting Items for Systematic Reviews and Meta-Analyses Protocols” (PRISMA) ^27^ and recently registered in PROSPERO ( CRD42020187469, 25 ^th^ June 2020). The completed PRISMA-P checklist for our protocol is included as *Extended data* ^28^. The ABI systematic review will be conducted and reported in accordance with the PRISMA guidelines, the National Institute for Health and Care Excellence (NICE) and the Cochrane Collaboration and 29. Any changes to the ABI systematic review protocol will be documented in the final published report.

### Search methods for identification of studies

***Search strategy.*** A systematic ABI literature research will be undertaken to identify relevant articles published until 24 ^th^ August 2020 in the following electronic databases: PubMed, MEDLINE (Ovid), EconLit , CINAHL (EBSCO), Embase, Web of science, NHS Database (NHSEED).

***Search terms.*** The search strategies will include Medical Subject Heading (MeSH) terms and key text words. The following MeSH terms and keywords will be used on their own or in combination: ‘Acquired brain injury’, ‘cost benefit’ , ‘cost’, ‘cost effectiveness’, ‘cost utility’, ‘economic evaluation’. The detailed search strategy is provided as *Extended data* ^28^. Retrieved search results will be downloaded into Endnote x9.3.1.

### Study selection procedure

Following the search, selected ABI studies will be independently screened according to the inclusion criteria by three reviewers, this initial search screening will be based on the study title and abstracts. A third reviewer will be consulted should there be any disagreement and will be responsible for a final decision. The PICOS criteria will be followed for the exclusion and inclusion of each ABI study. The PICOS is an acronym term that refers to the Population, Intervention, Comparison, Outcomes and Study design of an ABI article (see *Extended data* ^28^). Studies that may meet the agreed inclusion criteria will be examined in full and their details imported into in the EndNote x9.3.1 web library database.

The search and selection processes for this review will be displayed in a PRISMA flow diagram, including the results from the search, removal of duplicate citations, phases of studies selection (title/abstract and full text), the main reasons for excluded papers after full-text read. Endnote x9.3.1 and Covidence 2020 will be used to keep track of all references. Both stages of the selection process will be piloted on Covidence software.

### Inclusion and exclusion criteria

We will include studies that report cost-effectiveness analysis (e.g., cost per ABI life year gained), cost-benefit and cost minimisation analyses in which the main study designs were randomised controlled trials (RCTs), non-RCT studies, cost-utility analyses (e.g., cost per quality-adjusted life year (QALY) gained or cost per disability-adjusted life year averted), cohort studies, and modelling studies. Cost of illness analyses, narrative reviews, research protocols or conference abstracts will not be included. Additionally, studies on cadavers or animals will be excluded from this review. Studies in which there is no comparator group (i.e. usual care) will also be excluded.

The following PICO statements for a systematic search strategy will be used in this review- population (P), intervention (I), comparison (C), and outcomes (O):

**Population:** The population of interest will be individuals over 18 years old who have any type of acquired brain injury.

**Intervention:** Economic evaluation studies of non-pharmacological rehabilitation intervention for people with an ABI. We define non-pharmacological interventions as individual treatment not including drugs.

**Comparator:** Non-pharmacological rehabilitation intervention that stated a comparator intervention such as usual care or treatment as usual.

**Outcomes:** Incremental cost-effectiveness ratio (‘ICER’), incremental net monetary benefit (‘iNMB’), incremental net health benefit (‘iNHB’), incremental cost-utility ratio (‘ICUR’), and the incremental cost-benefit ratio. Secondary outcomes for this review will include: health related quality of life measures (HQol) used to calculate utilities (EuroQol, EQ-5D; SF-36, SF-6D) and intervention costs; costs arising from workforce productivity loss as a result of morbidity and mortality.

Table 1 displays the PICOS criteria for the ABI economic review

**Table 1.** PICOS criteria for the ABI economic review.

Criteria	Inclusion	Exclusion
Population	The population of interest will be individuals over 18 years old who have a mild, moderate, and/or severe type of acquired brain injury The term acquired brain injury (ABI): describes any injury sustained to the brain since birth. The Royal College of Physicians ^30^ have defined ABI as an inclusive category of injuries that embraces acute (rapid onset) brain injury of any cause, including: - trauma due to having a head injury (traumatic brain injury, TBI), or - vascular accident (sub-arachnoid haemorrhage or stroke), - metabolic or toxic insult (e.g. hypoglycaemia) - post-surgical damage (e.g. following a brain tumour removal), - cerebral anoxia, - other inflammation (e.g. vasculitis), or - infection (e.g. encephalitis, meningitis) Commonly, brain injuries can be sustained traumatically (TBI) following: - motor vehicle or road traffic accidents, - falls, or - assaults, or, according to ^31^ can be the result of a non-traumatic cause, such as : - stroke, - brain illness or tumor, - among other conditions There will be no restrictions on ABI participant characteristics such as age, gender, severity of acquired brain injury, study setting or country.	Interventions involving only children aged under 18 years of age will be excluded, due to the brain not being fully mature before this age, therefore being unable to rule out natural development versus recovery. Populations with mild cognitive impairment or other neurological disorders not related to ABI (i.e. dementia), will be excluded.
Interventions	Non-pharmacological rehabilitation intervention for people with an ABI (e.g., any treatment not involving drugs)	Pharmacological interventions that involve the consumption of a substance (including drugs, food supplements, herbal medicines, vitamins and homeopathic remedies.
Comparators	Usual care (UC) or treatment as usual (TAU).	Interventions that did not state a comparator intervention.
Outcomes	Patients: -Cost outcomes: Costs from healthcare and/or societal perspective; intervention costs; productivity loss costs due morbidity and mortality -Health outcomes: Health related quality of life (HQOL) measured as quality adjusted life years (QALY) by any instrument (e.g.: SF-6D or EQ-5D) or quality of life (QOL) measured by any instrument and disease specific health outcomes such as: days of institutionalized delayed, time to care home admission, hospital admission, etc. Care givers: -Cost outcomes: Heath-related cost or, productivity loss due to caregiving -Health outcomes: HQOL or QOL or psychosocial measures of caregiver burden etc. Results: -Net monetary benefit (NMB) -Incremental cost-effectiveness ratio (ICER) -Net health benefit (NHB)	Other than listed under inclusion criteria.
Study designs	Cost-effectiveness analysis (e.g., cost per life year gained), cost-benefit and cost minimization analyses in which the designs were randomised controlled trials (RCTs), non-RCT studies, cost-utility analyses (e.g., cost per quality-adjusted life year (QALY) gained or cost per disability-adjusted life year averted), cohort studies, and modeling studies.	Cost of illness studies (Col), editorials or other descriptive studies (e.g., protocols, single case reports) will be excluded.
General	Studies conducted anywhere in the world will be included, but only English language sources will be consulted. No date restrictions will be applied: sources will be searched from inception up to 24 ^th^ August 2020.	Non-English language studies

### Public and patient involvement

The general public and patients with an ABI will not be involved in this systematic review.

### Data collection and management

The literature search results will be managed using EndNote x9.3.1 to assist in the removal of duplicate records, recording decisions, study selection and references. data extraction and study selection and will be performed in Covidence and Microsoft Excel.

### Data extraction

The retrieved studies will be assessed in one of two phases; firstly, abstracts and titles will be checked, according to PICOS, and thereafter, the full text of the remaining ABI articles will be screened for final selection. Publication information, ABI study characteristics and findings from the included economic evaluation studies, related to the research question, will be recorded in a standardised, pre-piloted data extraction form using Microsoft Excel by two authors (EM and EA). Authors of studies will be contacted via e-mail, should there be missing data that is needed to be retrieved. Extracted information will include the following: (1) record details (author, title, publication date, journal); (2) study characteristics (trial design, country, sample size, analytical technique, population, intervention and comparator names and descriptions, primary clinical and economic outcome measure, time horizon, study perspective, cost categories, currency, price year); (3) study results (mean costs, mean effects, incremental costs, incremental effects, summary measure of efficiency (e.g. ICER, NMB). Information describing the intervention and comparator groups (including type, frequency and duration) will also be added to the data extraction sheet after the review has started.

Extracted information will include:

- Authors details
- Study title
- Publication year of ABI study
- Country of origin
- Objectives of the study
- Currency unit
- Study design
- Setting
- Target population
- Study perspective
- Sample size
- Intervention title and description
- Comparator title and description
- Measures of effectiveness
- Model specification
- Follow up duration
- Time horizon
- Description of resources used
- Price year
- Cost categories
- Discount rate
- Total/average intervention costs
- Total/average comparator costs
- ICER
- NMB or NHB
- Uncertainty analysis
- Sensitivity analysis
- Funding source

To validate the data extraction process, this process will be independently checked for completeness and accuracy by a third reviewer (SS). For this evaluation, the form will be structured based on the guidelines used to produce structured abstracts of economic evaluations for inclusion in the Consolidated Health Economics Evaluation Reporting Standards (CHEERS)” statement ^29^. Two reviewers will independently apply the checklist to the included studies.

### Analysis of subgroups or subsets

Subgroup analysis will only be considered where deemed appropriate.

### Assessing the quality of economic evidence

Two reviewers will use the CHEERS checklist ^29^, a tool recommended in a systematic review of tools. To validate the quality assessment process, a third reviewer will independently check the review. The CHEERS guideline has 24 items in six categories (title and abstract, introduction section, methods section, results section, discussion section and finally other). The items in the checklist were scored as ‘Yes’ (reported in full), ‘No’ (not reported at all), ‘PR’ (partially reported) and ‘Not Applicable’. In order to allocate a score of reporting, we will assign a score of 1 if the requirement of reporting was completely fulfilled for that item and 0 otherwise. Therefore, the highest maximum score that can be attained is 24. Any disagreements that may arise during this review stage will be resolved through discussion or will be addressed by a third reviewer (SS).

### Data analysis and synthesis

Following data extraction, the main results of the included studies will be displayed and summarized using tables. The structured summary will include information such as the country of publication, type of intervention treatment, type of ABI, type of economic evaluation and any other methodological features discussed among the included studies. The main methodological characteristics of included ABI studies will be summarized by using a “Characteristics of included ABI studies” table. The main findings of the review will enable discussion about future research, practice and policy for the ABI context. A tabulation of available unit cost data will also be completed. The currency and price year for each study, will also be reported. Where possible, incremental costs and cost-effectiveness data will be converted to 2020 British sterling value using implicit price deflators for GDP and GDP Purchasing Power Parities as suggested by CCEMG ^32^ to make relative comparisons between the effectiveness of studies in different countries.

### Reporting

This ABI systematic review and its findings will be reported in accordance to the PRISMA guidelines ^27^. The main implications of the ABI economic review findings will be discussed within the context of current and future policy related to ABI.

### Dissemination of findings

The findings of this systematic review will be disseminated through peer-reviewed publication. Additionally, findings will be presented at both national and international conferences and via a Public and Patient Involvement group of adults living with an ABI.

### Study status

The original search and first update search were conducted on 15th April and 20th July 2020 and 24 ^th^ August respectively. We have completed the initial screening process and are in the process of currently beginning to data extract information from selected studies.

## Discussion

Having an ABI not only result in long-term health effects, but also significant economic and social repercussions on individuals, their families, health-care providers and healthcare systems. Therefore, it is of vital importance for all stakeholders dealing with ABI treatment interventions to make effective and cost-effective health care decisions at an individual, societal, and international level. To the best of our knowledge, this is the first ABI systematic review that aims to review the economic evidence on non-pharmacological rehabilitation interventions. The goal of this review is to provide a reference source to help policy makers, healthcare professionals and academic researchers on how ABI services should be managed and assessed. The economic evidence from this review will be the most comprehensive review of economic evidence to date across ABI populations.

## Data availability

### Underlying data

No underlying data are associated with this article.

### Extended data

Zenodo: Extended data file - Neuropsychological rehabilitation interventions for people - A protocol. https://doi.org/10.5281/zenodo.4088035 ^28^.

This project contains the following extended data within the ‘Additional files.docx’:

- Additional file 2 – Search strategy
- Additional file 3 – PICO criteria

### Reporting guidelines

Zenodo: PRISMA-P checklist for “Neuropsychological rehabilitation interventions for people with an acquired brain injury. A protocol for a systematic review of economic evaluation”. https://doi.org/10.5281/zenodo.4088035 ^28^.

Data are available under the terms of the Creative Commons Attribution 4.0 International license (CC-BY 4.0).
