# Supplementary material for: Neuropsychological rehabilitation interventions for people with an acquired brain injury. A protocol for a systematic review of economic evaluation
Source: HRB Open Res. 2020 Dec 8;3:83. Originally published 2020 Nov 12. [Version 2] doi: 10.12688/hrbopenres.13144.2 (PMC7684672; doi:10.12688/hrbopenres.13144.2)
Supplement: Peer review history

# Peer review: Reviewer response for version 2

**DOI:** 10.21956/hrbopenres.14326.r28525

**Published:** 2020-12-10

**Authors:** Zartashia Ghani

**Affiliations:** 1 Department of Health, Blekinge Institute of Technology, Karlskrona, Sweden

No further comments.

Is the study design appropriate for the research question?

Yes

Is the rationale for, and objectives of, the study clearly described?

Yes

Are sufficient details of the methods provided to allow replication by others?

Yes

Are the datasets clearly presented in a useable and accessible format?

Not applicable

Reviewer Expertise:

My area of research is related to economic analysis, gerontechnology and mHealth applications.

I confirm that I have read this submission and believe that I have an appropriate level of expertise to confirm that it is of an acceptable scientific standard.

# Peer review: Reviewer response for version 1

**DOI:** 10.21956/hrbopenres.14258.r28354

**Published:** 2020-11-23

**Authors:** Lorcan Clarke

**Affiliations:** 1 Trinity College Dublin, Dublin, Ireland

This is a well put together protocol for a systematic review on an important topic, which the authors have clearly put structured thought and effort into.

 The only notable point of authors to consider is whether they are looking at "individuals over 18 years old who have any type of acquired brain injury." for their study population or, as outlined in their Table 1 and elsewhere in the manuscript "the population of interest will be individuals over 18 years old who have a mild, moderate, and/or severe type of acquired brain injury and/or caregivers to such a population." The authors need to clarify if caregivers are, or are not, part of the population considered for studies to be eligible for their review.

 During review, it was apparent that the protocol is comprehensive and may be benefit from refinement in some areas to ensure that the authors can complete the study process in a coherent and efficient fashion that ensures the users of this evidence are well informed by their findings. 

 The following comments follow the structure of the presented protocol:

 Abstract:

- Some of the aspects of the review (e.g. study type and search term) are specified in the abstract – but a lack of completeness may mean that the reader believes the authors have a conducted a narrower review than the authors intend to.
- It may be worth shortening the description of the inclusion criteria and search strategy to avoid this from happening.

Introduction

- Important to use the term “systematic review” throughout, rather than terms such as “economic systematic review”, “ABI systematic review” or “economic review”. Similarly, the term “protocol” should be used instead of “ABI economic evaluation protocol” or “studies” instead of “ABI studies”. Beyond clarity for the reader, the reason for this is that this protocol/review does not draw upon bespoke guidance for conducting such reviews/producing such protocols. (see Equator Network for examples of specific guidance on reviews in particular subject areas).
- The final introductory paragraph may benefit from focusing on one clear outcome of the review – e.g. “setting foundations to advance the field of health economic research for ABI-related interventions”, and then specifying the sub-activities within that goal.

Methods

- Search methods for identification of studies
  - Provide the exact date of searches (e.g. 1 June 2020 instead of June 2020, if that is the case).
  - Why did the authors use all the databases mentioned, is there a rationale which could be added?
  - Examples of search terms are provided, with the full complement noted as included in the “extended data” – it may be better to include all components of search strings or to simply include the categories of search terms within those search strings (e.g. general category for intervention, population, and study). The reason for this is that the search terms provided don’t reflect the full scope of the review as outlined by the authors.
- Study selection procedure
  - Typo – “acromion” (it’s part of your shoulder).
- Public and patient involvement
  - Typo – “The general public and patients with an ABI will not be involved in this overview of systematic reviews.” (the proposed study is a systematic review, rather than an overview)
- Data Extraction
  - There seems to be some inconsistencies between the proposed information for data extraction in the paragraph of the sub-section and the list provided after that paragraph.
  - Authors may want to review and consolidate the content into one single paragraph/list.
- Table 1
  - Why are caregivers included as the part of the study population or the subjects of the interventions? This inconsistency appears a couple of times in the protocol (inconsistent with description under population subheading on page 4/8). Important for authors to clarify if they are focusing on just the individuals with ABIs or including a focus on the support measures for their carers – if this is part of the focus, one could argue that a review of such (carer focused) interventions would benefit from having a wider scope than just carers for individuals with ABIs.
  - Use of term “non-pharmacological” may meant to be “non­-pharmaceutical” (term mix-up also appears in the main text).
  - Use of term “Non-English studies” should be “Non-English language studies”. Authors may benefit from outreach to experts in this research area who may have knowledge of the evidence base in other languages, and highlight briefly what exists in the discussion/limitations of their final manuscript. 
- Assessing Quality of Economic Evidence
  - CHEERS is useful for reporting of studies and thinking about the methods used, but authors may benefit from review of health economics literature which considers the reliability of health economic evidence (as GRADE does for clinical studies). For example check - https://www.ncbi.nlm.nih.gov/pmc/articles/PMC6743034/
- Data analysis and synthesis
  - Comparing studies by converting to equal monetary value (GBP 2020) has some benefit, but important for authors to recognise potential limitations, and study's quality and external validity differences, when conducting their comparison.

Is the study design appropriate for the research question?

Yes

Is the rationale for, and objectives of, the study clearly described?

Yes

Are sufficient details of the methods provided to allow replication by others?

Yes

Are the datasets clearly presented in a useable and accessible format?

Not applicable

Reviewer Expertise:

Economics, health economics, and health policy

I confirm that I have read this submission and believe that I have an appropriate level of expertise to confirm that it is of an acceptable scientific standard.

# Peer review: Reviewer response for version 1

**DOI:** 10.21956/hrbopenres.14258.r28355

**Published:** 2020-11-16

**Authors:** Zartashia Ghani

**Affiliations:** 1 Department of Health, Blekinge Institute of Technology, Karlskrona, Sweden

This paper reports a study protocol for a systematic review that will evaluate economic evaluations of neuropsychological rehabilitation interventions for people with an acquired brain injury and their caregivers. The rationale and objective for this review were clearly stated and search strategy is transparent in text. However, I have following comments and suggestions to improve this paper: 

1. Econlit is written twice in method section. Also, there are some other typos in the text which are needed to be corrected.
2. Study selection procedure (line7): *I think that instead of “…..acromion “ it should be “acronym”.*
3. Public and patient involvement: “The general public and patients with an ABI will not be involved in this overview of systematic reviews.”: *This is not clear. It seems that this study will be an overview of systematic reviews.*
4. Research studies within the time frame of last 5 or 10 years are usually considered updated, relevant or most useful. I think that it might be a good idea to discuss the reason/s behind not setting starting year for search strategy of this review in the main text.
5. In the process of systematic review writing, sometimes authors of included studies are needed to be contacted for further information. Thus, it might be a good idea to discuss the strategy, if required, how the authors of included studies will be contacted.
6. Scanning of reference list of selected studies and citations made on those selected studies can be useful to ensure that all the relevant studies are included. This process is sometimes referred as snowballing. I would like to suggest a good article entitled “ *Guidelines for Snowballing in Systematic Literature Studies and a Replication in Software Engineering* *https://www.wohlin.eu/ease14.pdf*” by Claes Wohlin for further guidance.
7. Additional file 1 – PRISMA-P Checklist: *Page numbers are needed to be revised.*

Is the study design appropriate for the research question?

Yes

Is the rationale for, and objectives of, the study clearly described?

Yes

Are sufficient details of the methods provided to allow replication by others?

Yes

Are the datasets clearly presented in a useable and accessible format?

Not applicable

Reviewer Expertise:

My area of research is related to economic analysis, gerontechnology and mHealth applications.

I confirm that I have read this submission and believe that I have an appropriate level of expertise to confirm that it is of an acceptable scientific standard.
